# Artificial intelligence–based calcium scoring using 3D transesophageal echocardiography in aortic stenosis: a pilot study

**DOI:** 10.1186/s44348-026-00080-x

**Published:** 2026-07-10

**Authors:** Paula Fazendas, Rita Bairros, Luís Brito Elvas, Liliana Brochado, João Carlos Ferreira, Rita Gomes, Ana Rita Pereira, Cristina Martins, José Pereira, Cândida Lourenço, Tomás Brandão, Hélder Pereira, Ana G. Almeida

**Affiliations:** 1 Department of Cardiology, Hospital Garcia de Orta, Almada, Portugal; 2 Cardiovascular Centre at the University of Lisbon (CCUL)–RISE, Faculty of Medicine, University of Lisbon, Lisbon, Portugal; 3 ISTAR, InstitutoUniversitário de Lisboa (ISCTE-IUL), Lisbon, Portugal; 4 Department of Logistics, Molde University College, Molde, Norway; 5 Inesc Inov Inovação – Instituto de Novas Tecnologias, Lisbon, Portugal; 6 Breast Cancer Research Program, Champalimaud Foundation, Lisbon, Portugal; 7 Department of Radiology, Hospital Garcia de Orta, Almada, Portugal

**Keywords:** Aortic valve stenosis, Artificial intelligence, Computer vision, 3D echocardiography, Calcification, Calcium score or scoring

## Abstract

**Background:**

Aortic valve calcium scoring by computed tomography (CT) is an established method for assessing aortic stenosis severity but is limited by radiation exposure and availability. Artificial intelligence (AI)-based calcium detection using transthoracic echocardiography has shown promise but depends on acoustic window quality. Transesophageal echocardiography (TEE), particularly 3D TEE, may overcome these limitations by providing improved visualization without radiation. The objective of this study is to evaluate the feasibility of AI-based quantification of aortic valve calcium using 3D TEE.

**Methods:**

In this prospective pilot study, 23 patients (median age, 76 years; 56.5% male) with moderate or severe aortic stenosis underwent 3D TEE and CT. Multiplanar reconstruction generated 1.5-mm diastolic short-axis slices. A computer vision–based model identified calcium-related speckles. An automated TEE calcium score was derived from the sum of calcium pixels across 11 frames per patient, which was compared with the CT Agatston score.

**Results:**

The TEE calcium score showed a significant positive correlation with CT Agatston scores (r = 0.65, P < 0.001). Receiver operating characteristic analysis yielded an area under the curve of 0.87 (95% confidence interval, 0.69–1.00) for identifying severe calcification (cutoff, 68,813 pixels; sensitivity, 89.5%; specificity, 75.0%).

**Conclusions:**

AI-based calcium quantification using 3D TEE is feasible and correlates with CT-derived scores. This radiation-free approach may provide a promising alternative for assessing aortic valve calcification.

## Introduction

Aortic stenosis (AS) is a common and progressive valvular heart disease, whose prevalence increases markedly with age [1, 2], affecting up to 12% of individuals over 75 years [3]. With the advancing longevity of the population, the burden of AS is expected to rise, making early detection and monitoring of the disease essential for improving patient outcomes.

While echocardiography is the primary tool for hemodynamic assessment, computed tomography (CT)-based aortic valve calcium scoring has emerged as a key independent predictor of adverse events and a refiner of risk stratification in uncertain cases [4–9]. However, the routine use of CT is limited by cost, availability, and, most importantly, ionizing radiation, which precludes its use for serial follow-up.

Transesophageal echocardiography (TEE) overcomes the limitations of poor acoustic windows. Specifically, 3D TEE allows for a comprehensive evaluation of the valve's calcium burden by identifying pixels across a larger portion of the aortic apparatus compared to 2D techniques. Despite this potential, clinical grading of calcium on ultrasound remains largely subjective.

According to current guidelines[], 3D TEE is indicated for assessing AS severity when transthoracic assessment is equivocal, as it provides accurate anatomical aortic valve area and left ventricular outflow tract measurements [4, 10]. It is also a cornerstone technique for transcatheter aortic valve implantation planning, allowing clinicians to obtain comprehensive anatomical data at a single time point [11]. For aortic valve planimetry, 3D TEE offers superior temporal resolution compared to cardiac CT [12–14], allowing for bedside, real-time evaluation that combines hemodynamic and anatomic data.

Artificial intelligence (AI), particularly deep learning and convolutional neural networks, is transforming echocardiography by enhancing image segmentation and automated measurements [15–17]. Recent models have successfully automated AS grading from 2D views and identified calcium pixels in both in vitro and in vivo studies [18–29]. However, current ultrasound-based AI models mostly rely on transthoracic imaging, which remains limited by image quality.

AI's ability to analyze large volumes of echocardiographic data holds great promise for improving diagnosis, enhancing disease monitoring and guiding treatment decisions in patients with AS.

We aimed to perform an exploratory study to evaluate the feasibility of an automated AI-based model to quantify aortic valve calcium burden using 3D TEE, with CT Agatston scoring as the reference standard.

## Methods

### Ethics statement

This study was approved by the Institutional Ethics Committee of Hospital Garcia de Orta, Unidade Local de Saúde Almada-Seixal (ULSAS), (No. 04/2024). All participants provided informed consent. The study protocol complied with the Declaration of Helsinki.

### Study population

This prospective pilot study included 23 consecutive patients with moderate or severe AS diagnosed by transthoracic echocardiography. Median age was 76 years (interquartile range [IQR], 73-84 15 years), and 56.5% were male. Patients were referred for TEE to assess AS severity due to poor TTE acoustic window, or to confirm associated multivalvular lesions. All patients underwent both 3D TEE and cardiac CT within a 3-month interval. Two control subjects without aortic valve disease were included to establish a baseline for calcium free image and methodological validation.

### Cardiac imaging

#### Echocardiography

**Fig. 1:**
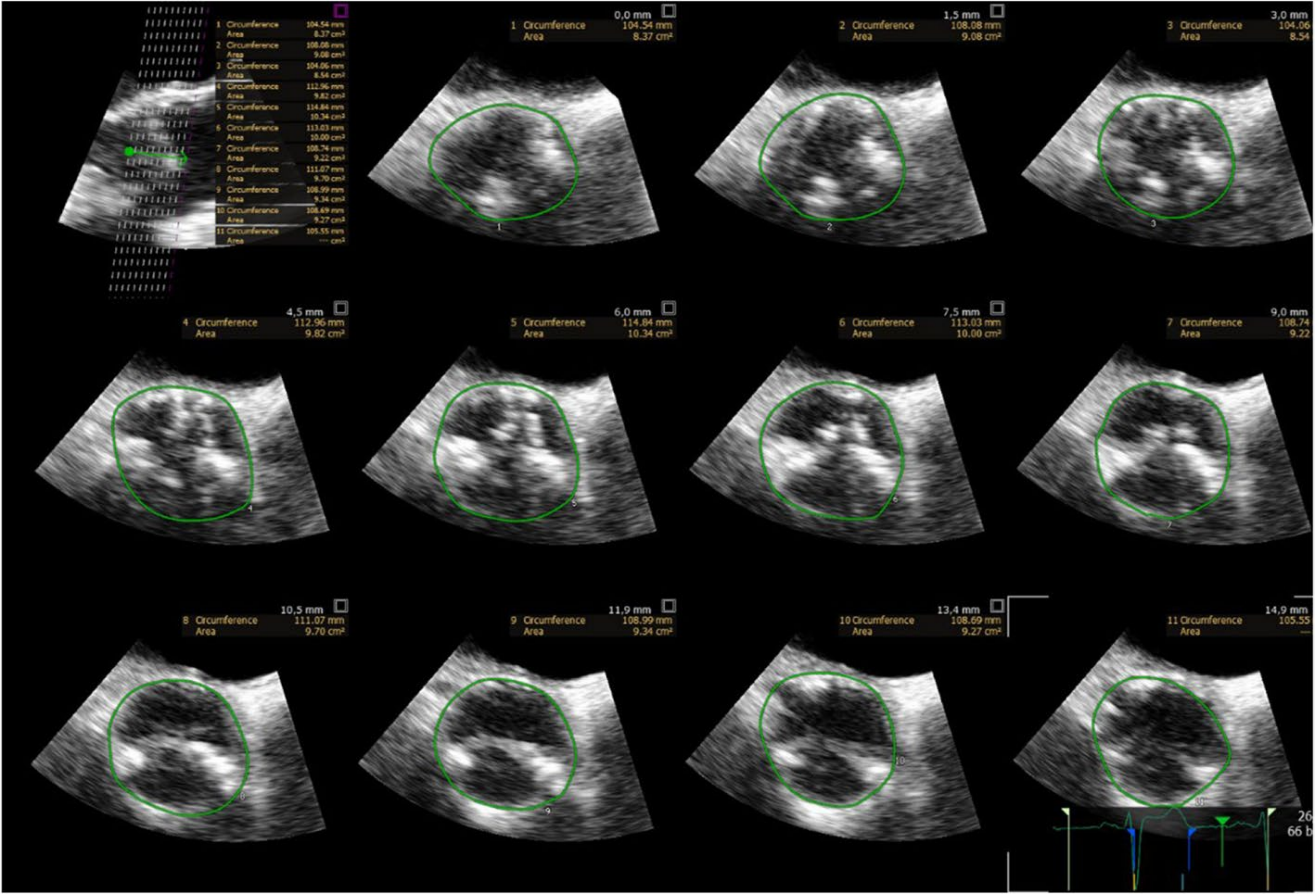
Multiplane rendering of the aortic valve in diastole depicting the intense echocardiography signals corresponding to zones of calcification at different valve levels

The 3D TEE studies were performed using a EPIQ system (Philips) with an X8T probe. Full-volume datasets of the aortic valve were acquired using 3D zoom and stored in DICOM format. Imaging parameters (gain, volume rate) and post-processing settings were kept constant across the entire cohort. Multiplanar reconstruction generated contiguous short-axis slices (1.5 mm thickness) in diastole, resulting in 11 images per patient. The region of interest (ROI), including the aortic valve cusps and annulus, was manually delineated (Fig. 1). Nonvalvular calcifications in areas such as the left ventricular outflow tract, aortic sinus, coronary arteries and mitral annulus were excluded from the analysis. A total of 253 DICOM images of AS patients were analyzed.

#### Cardiac CT

Noncontrast, electrocardiogram-gated multidetector cardiac CT was performed using 64-slice scanners, GE Revolution Evo (GE Healthcare) or GE Revolution Ascend (GE Healthcare), with a prospective protocol (120 kV, 3-mm thickness, triggered at 75% of the R-R interval). Aortic valve calcium scoring was calculated offline using the Agatston method via Smartscore 4.0 (GE Healthcare) or Vue PACS (Philips). Calcification was defined as density above 130 Hounsfield units within the aortic leaflets or annulus. Radiation exposure ranged from 1 to 1.5 mSv.

#### Data protection

All patient data, including clinical information and DICOM images, was anonymized to protect privacy. A research log was maintained, linking the anonymized data to a unique case number for future reference and comparison.

### Computer vision methods and AI-based quantification

A computer vision–based heuristic approach was developed to automate calcium quantification from 3D TEE images.

#### ROI extraction and pre-processing

**Fig. 2:**
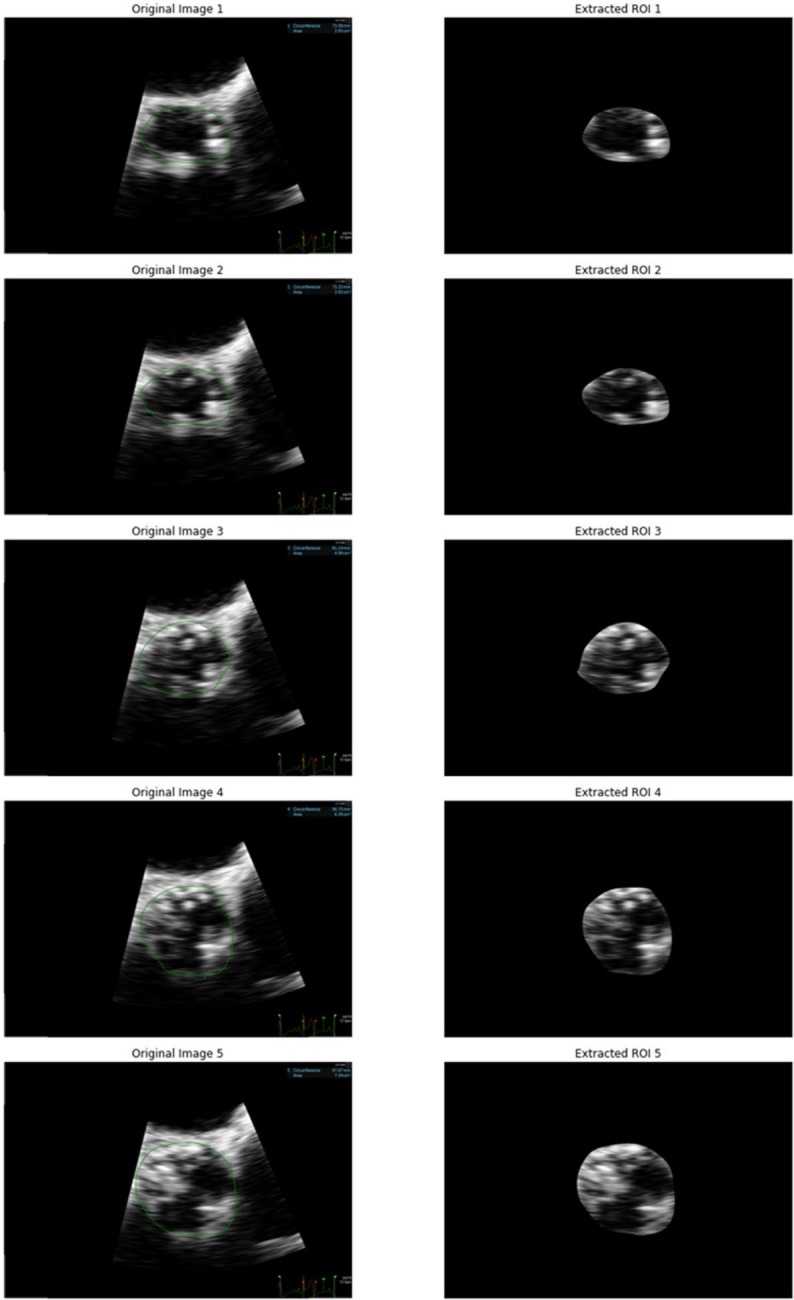
Original echocardiographic images and extracted region of interest (ROI)

The manually delineated ROI was isolated using a color-space filter HSV (hue, saturation, and value) to detect annotation markers and contour detection algorithms were applied to identify the edges of the ROI and isolate the aortic valve (Fig. 2). Images were subsequently cropped to the valvular apparatus to exclude nonvalvular noise.

#### Signal binarization and calibration

A thresholding technique was applied to distinguish calcium-related signals as white pixels. This allowed binarization of images into white (calcium) and non-white (not calcium). The thresholds were calibrated using the control subjects (without aortic valve disease) to ensure the absence of false-positive calcium pixels in noncalcified valves and to minimize background noise.

#### Automated TEE calcium score

**Fig. 3:**
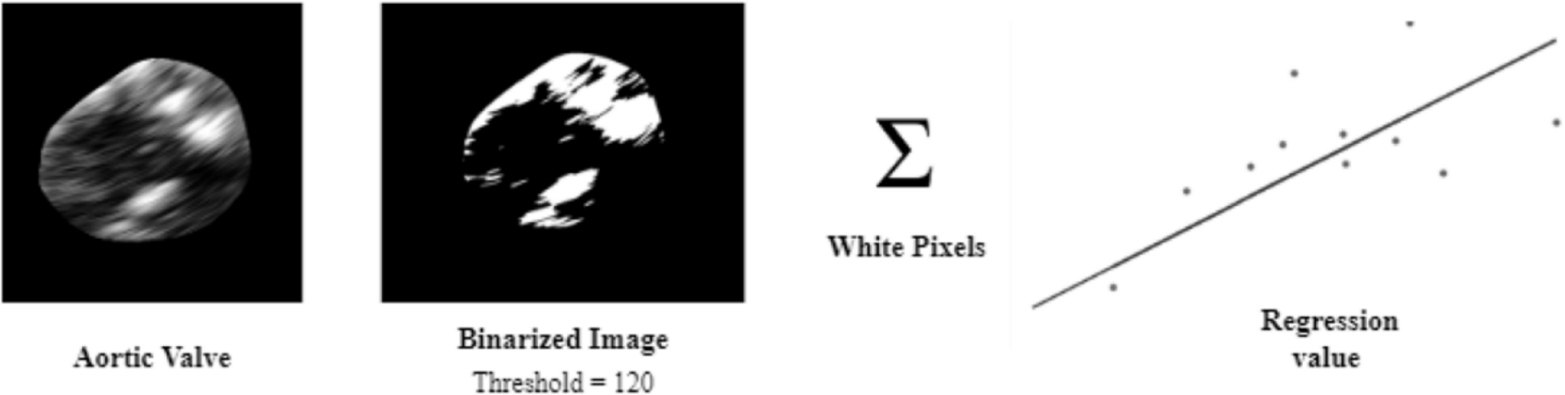
Framework for aortic valve isolation based in the manual traced region of interest and calcium scoring

The TEE calcium score was calculated as the cumulative sum of high-intensity white pixels (binarized signal) across 11 synchronized diastolic frames (Fig. 3). A range of intensity thresholds for binarizing the ROI images was evaluated to identify the one that most effectively correlated with the actual calcium scores obtained from clinical CT measurements. Different thresholds were systematically tested, and the corresponding calcium scores were calculated. To assess the strength and consistency of the relationship, three correlation methods were employed: Pearson, Spearman, and Kendall. The relationship between the TEE score and the CT Agatston scores, was explored using linear regression and regularized models (ridge and LASSO [least absolute shrinkage and selection operator]) to assess linear relations and reduce overfitting. Ensemble methods (random forest and gradient boosting) were also explored to assess the stability of the association and the potential for nonlinear relationships in cases of severe calcification.

### Clinical validation

To validate the effectiveness of the selected thresholds, binarized images were generated using the identified threshold range. These images were reviewed by two cardiologists specialized in imaging. The experts provided qualitative feedback on the clarity and accuracy of the binarized images to represent calcified regions. The cardiologists confirmed that the binarized images provided clear visual representations of potential calcifications, particularly in areas corresponding to known calcified regions on clinical CT scans.

### Statistical analysis

Population descriptives and comparisons between the two calcium scoring methods were expressed as mean ± standard deviation or median with IQR for continuous variables, depending on their distribution, and as percentages for categorical variables. Normally distributed variables were compared between the two patient groups using independent samples t-tests, with a 95% confidence interval (CI) level, assuming equal variances. Effect size Cohen d was adjusted using Hedges correction (Hedges g) for small sample sizes; an effect size of at least 0.5 was considered significant. Categorical variables were analyzed using the chi-square test for independence. In cases where expected cell counts were less than 5, Fisher exact test was applied to ensure valid results. Statistical significance was set at P < 0.05. Correlations between the two calcium scoring methods were assessed with linear regression models (Pearson r); Spearman and Kendall coefficients were used for nonlinear and ordinal associations, respectively. Receiver operating characteristic (ROC) curve analysis was used to determine cutoff values of the TEE Ca score for predicting severe calcification. All statistical analyses were performed using IBM SPSS ver. 28 (IBM Corp) and R ver. 4.5.2 (R Foundation for Statistical Computing) [30] within the RStudio integrated development environment ver. 2026.01.1 (Posit Software) [31].

## Results

### Baseline characteristics

The baseline characteristics of the study population are summarized in Table 1. A total of 23 patients were included (median age, 76 years [IQR, 73–84 years]; 56.5% male). Most patients presented with severe AS (82.6%) according to standard echocardiographic and Doppler parameters, with a median AVA of 0.73 cm^2^ (IQR, 0.62–0.94 cm^2^) and a mean pressure gradient of 42.4 ± 16.2 mmHg. Left ventricular systolic function was preserved in the majority of the cohort (median left ventricular ejection fraction, 63.9%; IQR, 56–66%). The mean interval between TEE and CT scans was 65 ± 47 days.

**Table 1:** Baseline patients’ characteristics (n = 23)

Characteristic	Value
Age (yr), median (IQR)	76 (73–84)
Sex (%)	
Male	13 (56.5)
Female	10 (43.5)
Body mass index (kg/m^2^)	27.86 ± 4.79
Body surface area (m^2^/kg)	1.82 ± 0.21
Left ventricle ejection fraction (%), median (IQR)	63.9 (56–66)
Aortic valve area (cm^2^) (continuity equation), median (IQR)	0.73 (0.62–0.94)
Mean aortic gradient (mmHg)	42.4 ± 16.2
Peak aortic velocity (m/sec)	4.19 ± 0.75
VTI ratio, median (IQR)	0.23 (0.20–0.26)
AS severity (%)	
Mild	1 (4.4)
Moderate	3 (13.0)
Severe	19 (82.6)
Aortic valve area (cm^2^) (3D planimetry)	0.88 ± 0.24

Values are presented as number (%) or mean ± standard deviation, unless otherwise indicated

AS, aortic stenosis; IQR, interquartile range; VTI, velocity time integral

### AI model calibration and threshold selection

**Fig. 4:**
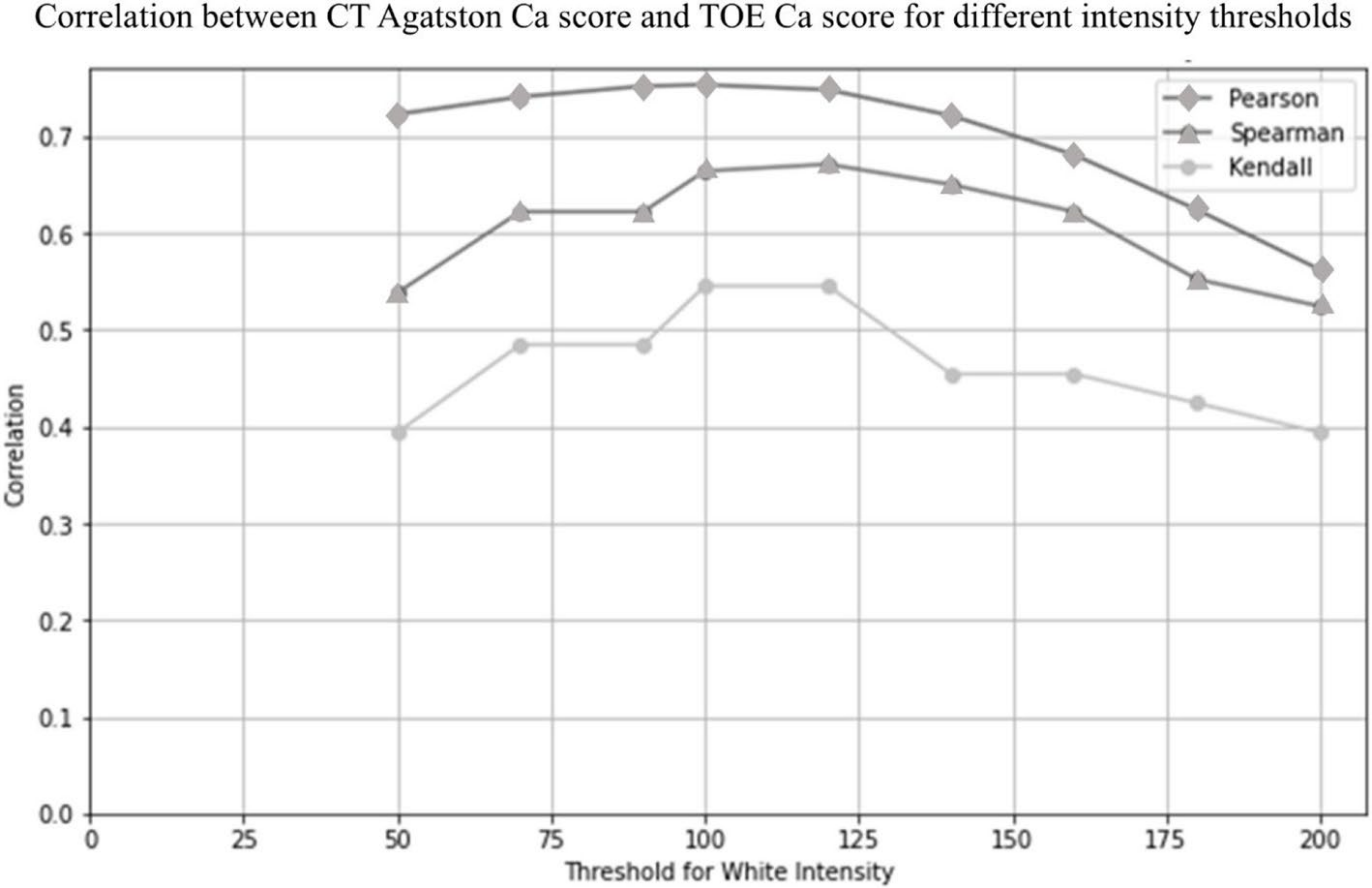
Correlation analysis of intensity thresholds. Pearson, Spearman, and Kendall correlation coefficients are plotted against varying intensity thresholds. CT, contrasted tomography; Ca, calcium; TEE, transesophageal echocardiography

**Fig. 5:**
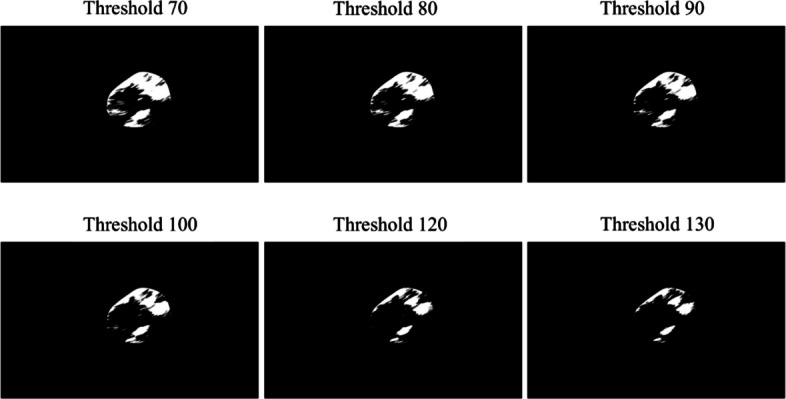
Binarized images for intensity thresholds 70–130

Correlation metrics for various intensity thresholds are illustrated in Fig. 4. Association metrics peaked at a threshold range between 100 and 120. Qualitative analysis of the binarized images by two expert cardiologists confirmed that an intensity threshold of 100 provided the best performance for identifying calcified regions while minimizing noise (Fig. 5).

### Correlation between TEE and CT calcium scores

**Fig. 6:**
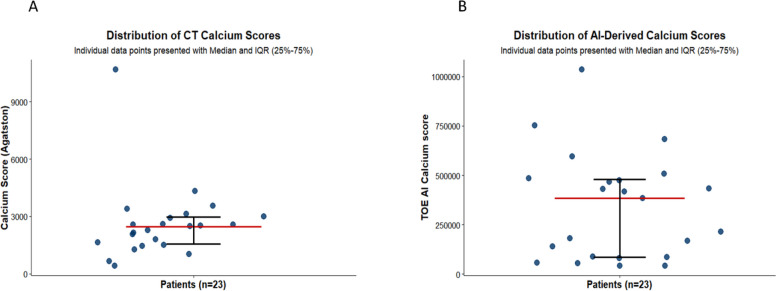
Scatter plots of individual patient data (*n* = 23). **A** Computed tomography (CT) calcium score and (**B**) transesophageal echocardiography (TEE) artificial intelligence (AI)-derived calcium score distribution. Statistical centrality and dispersion are represented by the median (red line) and interquartile range (black bars), accounting for the non-normal distribution of the scores

Comparative results for CT Agatston and AI-derived TEE calcium scores are presented in Table 2 and Fig. 6. The median Agatston score was 2,479 (IQR, 1,570–2,966), while the AI-derived TEE calcium score (at the 100-intensity threshold) was 385,203 pixels (IQR, 86,099–480,326 pixels).

**Table 2:** Descriptive statistics of calcium scores (n = 23)

Variable	Median (IQR)	Range (minimum–maximum)	P-value^a^
CT calcium score (Agatston)	2,479 (1,570–2,966)	421–10,679	< 0.001
TOE-AI score	385,203 (86,099–480,326)	41,573–1,035,608	0.025

Scores are expressed in Agatston units

AI, artificial intelligence; CT, computed tomography; TOE, transesophageal echocardiography

^a^Shapiro-Wilk test

**Fig. 7:**
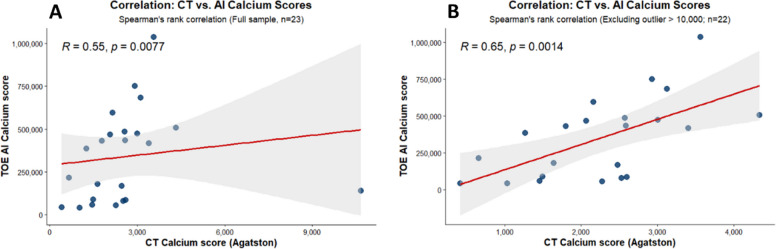
Predictive relationship of calcium scores. Correlation between computed tomography (CT) Agatston scores and transesophageal echocardiography (TEE) artificial intelligence (AI) TEEscores (n = 23). The regression line and 95% confidence interval are shown. **A** Correlation was assessed via Spearman rank analysis, yielding a significant predictive association (P = 0.041) across the observed clinical range. **B** Excluding the outlier

TEE A significant positive correlation was observed between the two methods (Spearman R = 0.55, P = 0.008) (Fig. 7A). One patient with an extreme CT Agatston score (10,679), confirmed by independent evaluation from a cardiologist and a radiologist, was identified as an outlier. To assess the impact of extreme values, a sensitivity analysis was performed by excluding the outlier (CT score > 10,000). This led to a strengthened association, with the Spearman correlation coefficient increasing to 0.65 (*P* = 0.001) (Fig. 7B), confirming that the correlation remains robust across the standard clinical range.

TEE

### Diagnostic performance and ROC analysis

**Fig. 8:**
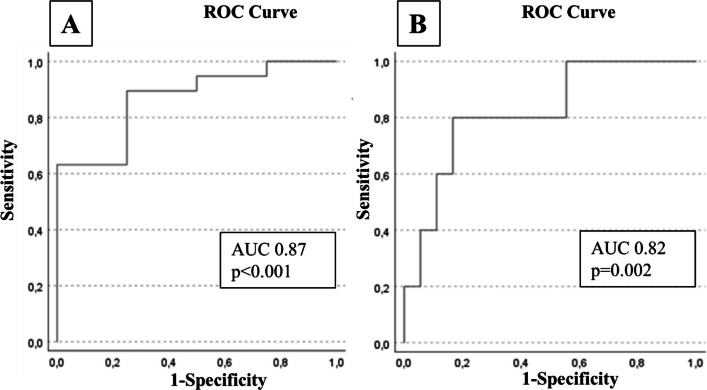
Receiver operating characteristic (ROC) curve analyses of the transesophageal echocardiography (TEE) calcium TEE score to detect (**A**) “very likely” or “likely severe calcification” and (**B**) “very likely severe calcification” as defined by the aortic valve Agatston calcium score

ROC curve analysis demonstrated high diagnostic accuracy for the TEE calcium score in identifying severe calcification, with an area under the ROC curve (AUC) of 0.87 (95% CI, 0.69–1.00) (Fig. 8A). A cutoff value of 68,813 pixels yielded a sensitivity of 89.5% and a specificity of 75.0%. The model maintained excellent performance when detecting "very likely" severe calcification, with an AUC of 0.82 (95% CI, 0.54–0.95) for a cutoff of 471,062 pixels (sensitivity, 80.0%; specificity, 83.3%) (Fig. 8B). Notably, this AI cutoff successfully identified the AS patient with extreme calcification as “very likely severe.”

TEE

## Discussion

This pilot study demonstrates that an AI-based computer vision model can accurately identify and quantify aortic valve calcification using 3D TEE, showing a significant correlation with the gold-standard CT Agatston score. While echocardiography remains the primary tool for evaluating AS severity, traditional grading of calcification has been limited to subjective, qualitative assessment. Our findings suggest that automated, pixel-based quantification could bridge this gap, offering an objective, radiation-free alternative. By establishing the feasibility of an AI-derived TEE calcium score, this study serves as a proof of concept, providing the necessary evidence to scale this approach in future, more extensive investigations.

Initially, the entire cohort yielded a moderate correlation. However, the presence of an extreme outlier (CT score > 10,000) warranted closer inspection. A sensitivity analysis excluding this single data point improved the correlation coefficient to moderate-to-strong (R = 0.65), demonstrating that the predictive accuracy of the AI algorithm is even higher within the standard clinical range of cardiac calcification. The exclusion of extreme outliers is a recognized statistical practice in pilot studies with small sample sizes, as it prevents a single disproportionate value from skewing the overall trend. In this case, the extreme outlier likely represents a distinct clinical phenotype of massive calcification, where acoustic shadowing in TEE might affect measurements. Our findings suggest that while the TEE-AI tool is capable of reflecting extreme scores, its reliability and precision are optimal for the majority of patients who present with typical levels of calcium burden. This strengthens the argument for the clinical utility of AI-enhanced TEE as a screening tool, particularly when extreme values are managed with specialized clinical oversight.

Although massive acoustic shadowing mathematically compromised the model’s ability to output a perfectly proportional absolute score—leading to its exclusion from the continuous linear analysis—the AI algorithm successfully and correctly categorized this patient as having "very likely severe calcification.” Therefore, this limitation is strictly mathematical in terms of absolute quantitative alignment in extreme values, rather than a clinical failure in diagnostic categorization.

Furthermore, TEE inherently minimizes acoustic shadowing compared to transthoracic approaches because it utilizes higher frequencies made possible by the short anatomical distance to the aortic valve [12], thereby offering superior spatial resolution. In clinical practice, slightly adapting the imaging plane also allows operators to overcome acoustic shadowing obstacles.

### Clinical implications

The potential for practical clinical application is significant. By providing an automated, quantitative score during a standard 3D TEE, this method could streamline workflows and improve risk stratification. Patients with intermediate or discordant echocardiographic findings and high AI-derived calcium scores could be prioritized for further intervention or CT confirmation. The primary clinical utility of our AI tool lies in opportunistic screening. By applying this software to images already acquired for routine clinical indications (e.g., preprocedural planning, evaluation of masses, or exclusion of thrombi), clinicians can obtain valuable calcium scoring data as a "byproduct" without exposing the patient to additional radiation, costs, or iodinated contrast media.

Considering the semi-invasive nature of TEE, large multicenter data demonstrates it is exceptionally safe when performed by experienced operators [32], with a significant complication rate of less than 0.02% in over 10,000 procedures. Conversely, while multidetector CT is noninvasive, it carries risks such as contrast-induced acute kidney injury, which can occur in up to 10% of high-risk patients such as the elderly AS patients [33].

### Limitations

Several limitations should be addressed. First, the small sample size requires further validation in larger, multicenter cohorts. While deep learning models were explored in this pilot phase, the modest sample size favored a heuristic computer vision approach to ensure clinical transparency and statistical robustness. Although the population studied is small, our data involved the analysis of 253 independent frames systematically extracted from multiple spatial planes of real-time 3D volume cineloops. Because these frames capture the aortic valve from multiple, distinct spatial planes within the 3D volume, they provide a highly rich and heterogeneous dataset. From a computer vision validation perspective, this frame-level sample size (n = 253) provides a solid statistical and technical foundation for an initial pilot validation.

Second, the current model requires manual identification of the ROI, which introduces potential user variability. Future developments focusing on automated deep learning ROI detection, valve segmentation, and quantification [34] could enhance reproducibility and reduce post-processing time. Third, we noted a mean delay of 65 days between TEE and CT scans. However, AS is typically an indolent disease. With an average annual Agatston progression of approximately 158 units [35], a 3-month window would result in a negligible difference of roughly 40 units—well within the technique's variability [36]. The strong performance observed despite this delay further supports the robustness of the TEE-based score.

### Challenges and future directions

Translating AI tools into clinical practice involves challenges beyond technical accuracy, including regulatory approval, data privacy, and integration into existing electronic health records. Ensuring the interpretability of AI-driven decisions is also crucial for building clinician trust. Future research should explore broader ranges of AS severity and investigate the automatic recognition of calcium-induced artifacts as additional diagnostic markers, as well as expand our model to include multivalvular assessment such as the quantification of mitral annular calcification.

## Conclusions

AI-based quantification of aortic valve calcium using 3D TEE is feasible and shows significant preliminary correlation with CT Agatston scores. This radiation-free approach warrants further validation as a potential tool for objective AS assessment and serial patient monitoring.

## Data Availability

No datasets were generated or analysed during the current study.

## Abbreviations

AI: Artificial intelligence
AS: Aortic stenosis
AUC: Area under the receiver operating characteristic curve
CI: Confidence interval
CNN: Convolutional neural network
CT: Computed tomography
HSV: Hue, saturation, and value
IQR: Interquartile range
LASSO: Least absolute shrinkage and selection operator
ROC: Receiver operating characteristic
ROI: Region of interest
TEE: Transesophageal echocardiography
TOE: Transoesophageal echocardiography
